# A dedicated beamline for wide-energy-range X-ray spectroscopy at SSRF: combining soft and hard X-ray capabilities

**DOI:** 10.1107/S1600577525011506

**Published:** 2026-01-30

**Authors:** Zhaofeng Liang, Jinyang Xu, Lei Xie, Jingyuan Ma, Bingbao Mei, Liangxin Wang, Nan Wang, Zhenhua Chen, Ying Zou, Fei Song

**Affiliations:** a Shanghai Synchrotron Radiation Facility Shanghai Advanced Research Institute Shanghai201000 People’s Republic of China; b University of Chinese Academy of Sciences Beijing100100People’s Republic of China; ESRF – The European Synchrotron, France

**Keywords:** canted undulators, combined soft/hard beamline, HAXPES, near-ambient pressure, XAS, X-ray absorption spectroscopy, photoemission spectroscopy

## Abstract

The advancement of renewable energy critically depends on the rational design of catalysts, which necessitates a thorough understanding of the underlying materials. At Shanghai Synchrotron Radiation Facility (SSRF), the Energy Material beamline (E-line) has been established with three independent endstations – soft X-ray, hard X-ray and a combined soft/hard station – dedicated to fundamental studies of photovoltaic and catalytic processes, energy conversion mechanisms and related phenomena. Covering a broad photon energy range from 130 eV to 10000 eV, the combined endstation is specifically designed for *in situ* photoemission spectroscopy, enabling layer-by-layer analysis of materials and devices. Through techniques such as wide-range hard X-ray photoemission spectroscopy (HAXPES) and X-ray absorption spectroscopy (XAS), this endstation provides comprehensive insights into the chemical and electronic properties of catalysts. This report outlines the layout of the combined soft/hard beamline and the endstation, and evaluates its performance in terms of photon flux, energy resolution and representative applications in model catalysis. In particular, the use of *in situ* X-ray photoemission spectroscopy/HAXPES is expected to significantly advance the fundamental understanding of functional materials, thereby accelerating the development of efficient, reliable and affordable renewable energy solutions.

## Abstract 2

A wide-range beamline (130–10000 eV) combining both soft X-ray and hard X-ray photons has been constructed at SSRF for *in situ*X-ray photoemission spectroscopy investigations of the solid–gas interface, and for hard X-ray photoemission spectroscopy studies with layer-by-layer resolution.

## 1. Introduction

Over the past decades, the continuous rise in fossil fuel consumption has led to a substantial increase in carbon dioxide emissions, contributing significantly to global warming and other adverse environmental impacts. In response, there is a growing demand for clean and renewable energy alongside a greener environment, which has driven research not only into noble-metal catalysts such as Pt and Ir, despite their high cost, but also into transition-metal-based catalysts (*e.g.* Fe, Co, Ni), which offer a promising alternative due to their cost-effectiveness (Ezhov *et al.*, 2024 ▸; Zeng *et al.*, 2023 ▸; Guo *et al.*, 2024 ▸). Furthermore, the development of high-performance and low-cost catalysts is closely related to elucidation of complex reaction mechanisms in the energy conversion and storage process (Yano & Yachandra, 2009 ▸; Cutsail & DeBeer, 2022 ▸; Rocha *et al.*, 2012 ▸). Fortunately, X-ray spectroscopy offers a powerful approach for determining both atomic structures and electronic structures of materials. Its element-specific ability enhances the probing of individual elements in complex systems without mutual interference (Xu *et al.*, 2025 ▸; Eggart *et al.*, 2021 ▸; Garcia-Diez *et al.*, 2025 ▸). By advancing fundamental insights through techniques such as X-ray absorption spectroscopy (XAS), X-ray emission spectroscopy (XES) and X-ray photoemission spectroscopy (XPS), a solid foundation will be established for developing more efficient, reliable and sustainable energy solutions.

In general, XAS/XES measures provide bulk-sensitive information, with penetration depths on the order of micrometres or more. In contrast, XPS is surface-sensitive, typically probing only the top few nanometres of the investigated material, as determined by the inelastic mean free path (IMFP) of photoelectrons (Abbate *et al.*, 1992 ▸; Frazer *et al.*, 2003 ▸). Importantly, it is widely recognized that most chemical reactions in energy conversion and storage processes occur at surfaces or interfaces, involving the adsorption of reactants, formation of intermediate species and desorption of products (Nørskov *et al.*, 2011 ▸; Ruiz-Lopez *et al.*, 2020 ▸). In practice, conventional soft X-ray XPS using Al/Mg *K*α sources or synchrotron radiation below 2000 eV has a probing depth of less than 10 nm. By contrast, hard X-ray photoemission spectroscopy (HAXPES), which employs photons with energies above 2 keV, can probe buried interfaces up to several hundred nanometres deep, as the IMFP of photoelectrons increases with kinetic energy above approximately 100 eV (Risterucci *et al.*, 2014 ▸; Woicik, 2016 ▸; Rueff *et al.*, 2018 ▸; Kalha *et al.*, 2021 ▸; Favaro *et al.*, 2021 ▸). Moreover, XPS has evolved into an accessible method for studying samples in various forms under *in situ* or *operando* conditions, such as in gas environments, and under varying temperatures or external electric fields. Given these capabilities, wide-energy-range XPS is now regarded as a versatile approach for revealing the atomic structure of materials under diverse *in situ* conditions. Consequently, several wide-energy X-ray beamlines have been constructed and are now operational worldwide, including the EMIL beamline at BESSY II, the X SPEC beamline at the KIT light source, the SST beamline at NSLS-II and the I09 beamline at Diamond Light Source (Hendel *et al.*, 2016 ▸; Weinhardt *et al.*, 2021 ▸; Lee & Duncan, 2018 ▸; Weiland *et al.*, 2018 ▸).

In this context, a wide-energy-range beamline, referred to as the Energy Material beamline (E-line), has also been proposed and constructed at the Shanghai Synchrotron Radiation Facility (SSRF), as introduced earlier (Chen *et al.*, 2018 ▸). The E-line consists of three branches: soft X-ray, hard X-ray and a combined soft/hard X-ray branch, which successfully entered commissioning in 2024 (Mei *et al.*, 2024 ▸). SSRF is a third-generation light source comprising a 150 MeV linear accelerator and a 3.5 GeV electron storage ring, operating in top-up mode with a beam current of 200 mA. Like other wide-energy-range beamlines, the E-line incorporates two independent canted undulators to simultaneously generate soft and hard X-rays. A distinctive feature, however, lies in the soft/hard combined branch, which is specifically optimized for wide-range XPS under *in situ* conditions. Accordingly, this article provides a detailed account of the performance of the combined soft/hard branch and presents initial experimental results obtained at its wide-energy-range endstation.

## 2. Description of the combined soft/hard beamline and its performance

**Figure 1:**
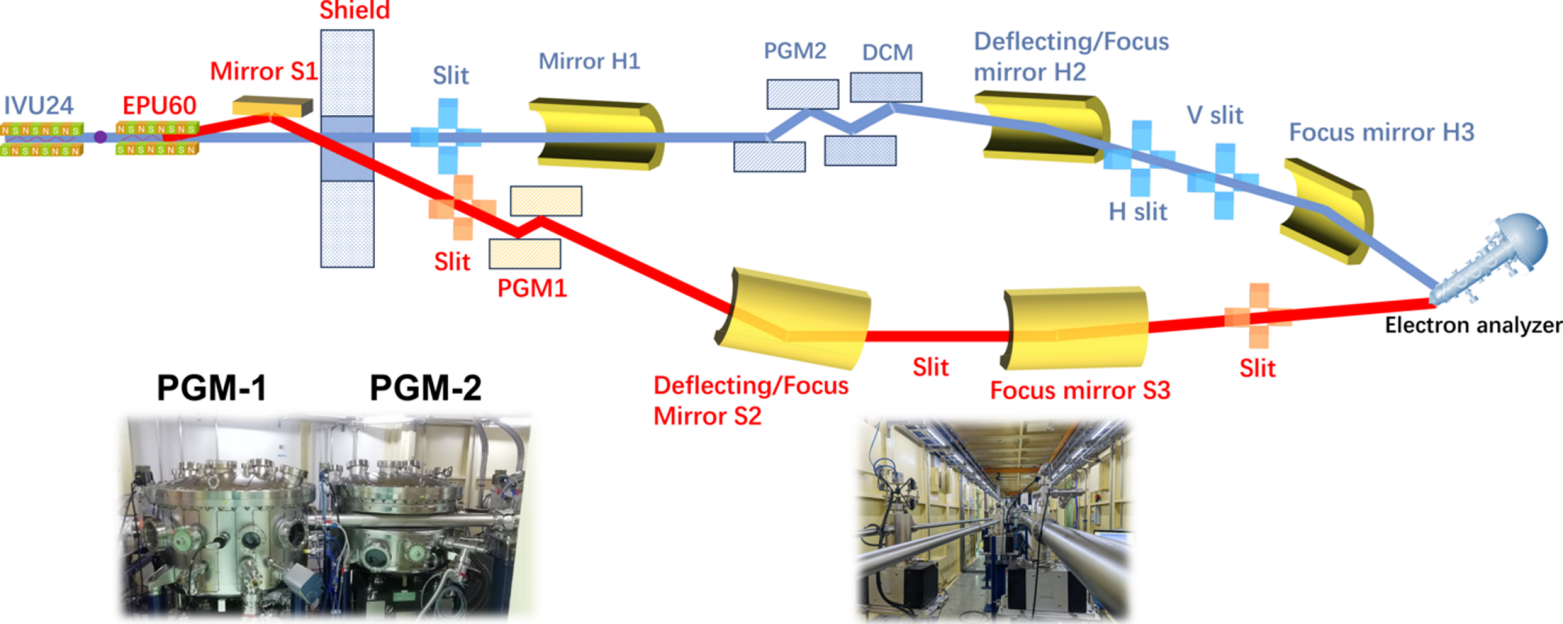
Diagram of the combined soft/hard beamline at SSRF and the corresponding photographs of PGMs, where the interweave of PGM-1 and PGM-2 is highlighted.

To deliver soft, tender and hard X-rays to the respective branches of the E-line, two independent undulators, an elliptical polarized undulator (EPU) and an in-vacuum undulator (IVU), are installed along the same straight section with a canted angle of 2 mrad, as shown in Fig. 1 ▸. The EPU covers the soft X-ray range from 130 eV to 1500 eV for the soft X-ray branch, which is equipped with resonant inelastic X-ray scattering (RIXS) and resonant elastic X-ray scattering (REXS). The IVU supplies tender and hard X-rays from 1500 eV up to 18 keV for the hard X-ray branch, which supports high-energy-resolution fluorescence detection X-ray absorption near-edge spectroscopy (HERFD-XANES) and resonant X-ray emission spectroscopy (RXES) (Chen *et al.*, 2018 ▸; Mei *et al.*, 2024 ▸). Key parameters of the EPU and IVU are summarized in Table 1 ▸; further details can be found in previous reports (Chen *et al.*, 2018 ▸; Mei *et al.*, 2024 ▸) and in Fig. S1 of the supporting information. As illustrated in Fig. 1 ▸, the soft X-ray beam (red line) is monochromated using a water-cooled variable-line-spacing plane-grating monochromator (PGM-1). PGM-1 employs blazed gratings with central line densities of 300, 800 and 1200 lines mm^−1^, similar to the in-focus variable-line-spacing PGM design reported earlier (Cai *et al.*, 2019 ▸). On the hard X-ray branch, most of the IVU beam (2500–18000 eV, blue lines) is monochromated by a double-crystal monochromator (DCM) using Si(111) and Si(311) crystals. The tender-energy portion (1500–2500 eV) is monochromated by a second plane-grating monochromator (PGM-2) with fixed groove densities of 800 and 1200 lines mm^−1^. Due to the extremely high heat load, PGM-2 is cooled with liquid nitro­gen. PGM-2 and the DCM are arranged in series along the hard X-ray branch. Either monochromator can be moved into or out of the beam path as needed, ensuring that only one is in the beam at a time.

To merge the soft and hard X-ray beams together, each beam is first deflected by its respective mirror: the soft-branch mirror S2 and the hard-branch mirror H2, as shown in Fig. 1 ▸. The soft X-ray beam is then refocused both horizontally and vertically by the toroidal mirror S3 into the combined endstation. Meanwhile, the hard X-ray beam is pre-focused horizontally by the toroidal mirror H1 onto the H-slit while remaining parallel in the vertical direction. The cylindrical mirror H2 subsequently deflects the hard beam into the combined branch and focuses it vertically onto the V-slit. Finally, as indicated in Fig. 1 ▸, a bent toroidal mirror H3 refocuses the hard X-ray beam into the combined endstation. The soft and hard beams are thus brought to a common focal point with a crossing angle of 2°, forming a new branch designated as the combined soft/hard beamline. Although the IVU can supply photons with energies beyond 10000 eV, the combined endstation is specifically configured for XPS studies. Consequently, the photon energy (*hv*) range is restricted to 130–10000 eV, due to the upper kinetic energy limit of the electron spectrometer and the very low photoionization cross section at higher photon energies.

**Figure 2:**
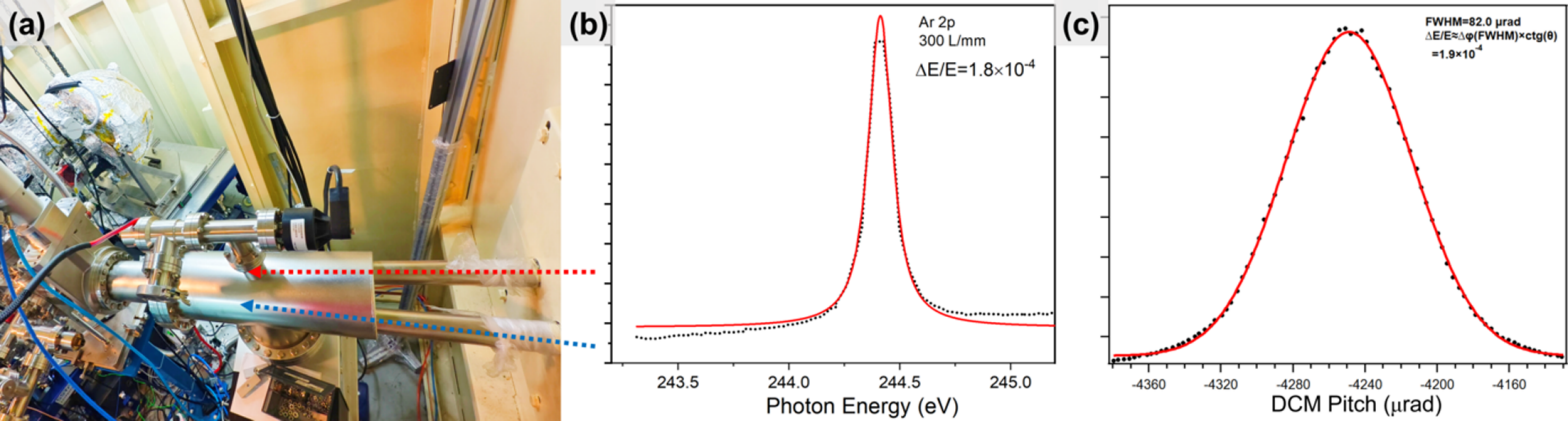
(*a*) Photograph of the combination of the soft X-ray (red) and hard X-ray (blue) into the combined endstation. (*b*) The resolution power (Δ*E*/*E*) of the combined beamline at 244 eV and (*c*) 5000 eV.

**Figure 3:**
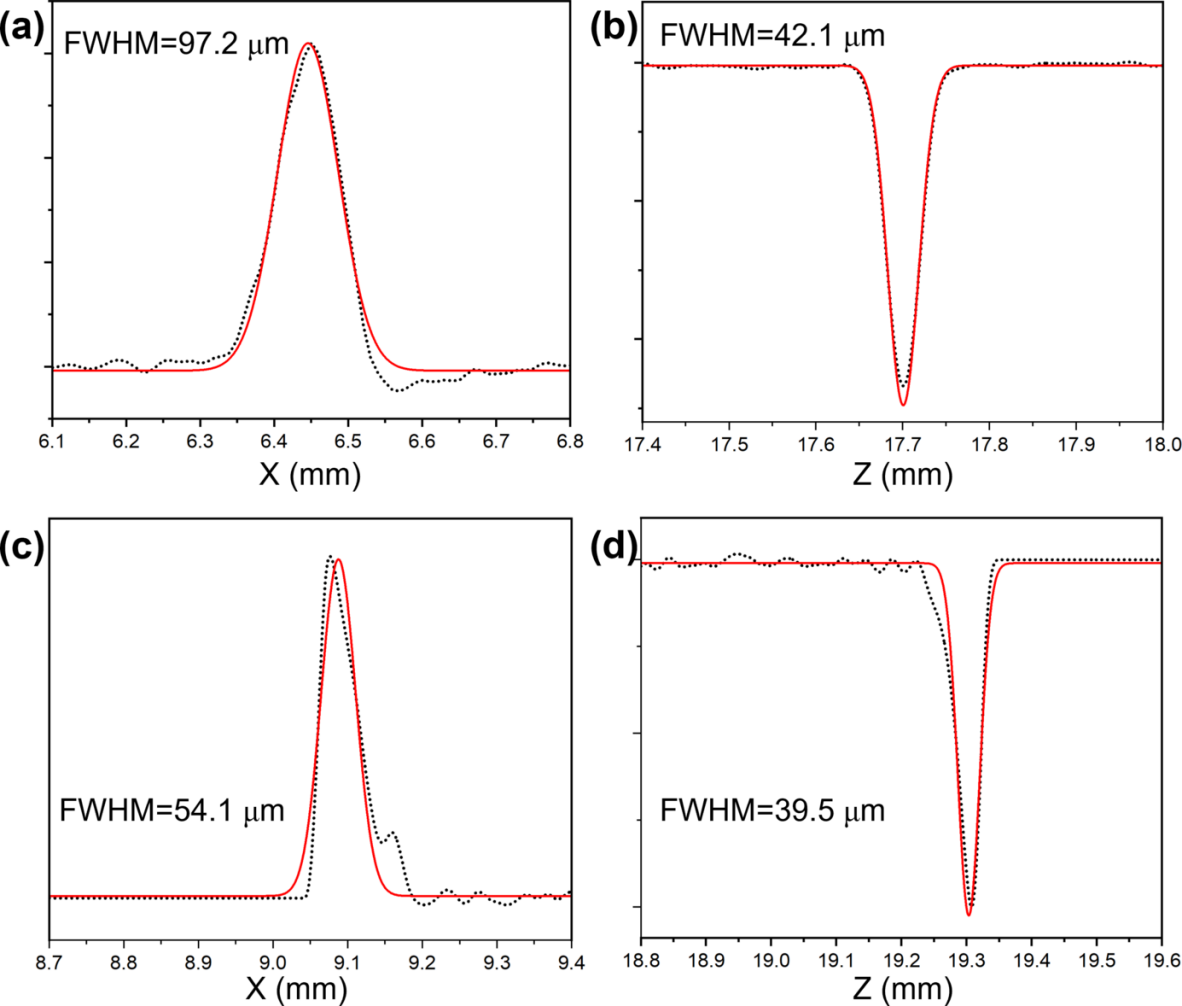
Measurement of beam spot sizes (Gaussian fitting of the first derivation) at horizontal (*a*) and vertical directions (*b*) at 244 eV, and at horizontal (*d*) and vertical directions (*d*) at 5000 eV.

The switching of the X-ray beam between the soft/hard branch and the combined branch can be achieved by linearly translating the S1 mirror or by shifting the cylindrical mirror H2. Notably, to effectively resolve the spatial conflict between the two PGMs, PGM-2 is integrated such that it is partially enclosed by the vacuum tube of PGM-1, as shown in the inset of Fig. 1 ▸. This unique design allows PGM-1, PGM-2 and the DCM to operate simultaneously without interference. With the combined branch configured as shown in Fig. 1 ▸, a wide-range X-ray beam is delivered to the combined endstation [Fig. 2 ▸(*a*)], and the resolution power of the combined beamline is first verified by measuring the broadening of the Ar 2*p*_3/2_ absorption spectrum at 244 eV (the *L*_3_ edge), and the rocking curve of the DCM at 5000 eV. In the hard X-ray regime, the energy resolution is primarily determined by the Darwin width of the given Bragg reflections in the DCM. According to the Bragg diffraction principle, the relative bandwidth Δλ/λ (or Δ*E*/*E*) is exactly given by Δθ × cot θ. θ is the Bragg angle at the chosen photon energy, and Δθ can be characterized by the rocking curve – a high-resolution X-ray diffraction technique that measures the variation in diffracted intensity as the crystal is gradually tilted (‘rocked’) relative to the incident beam, thereby providing a quantitative assessment of the resolution power. In addition, the choice of 244 eV and 5000 eV to test the resolution power is based on ray-tracing results. After subtracting the pristine Lorentzian broadening of the Ar 2*p* inner-shell ionization (the natural lifetime of the core-excited state) from the overall full width at half-maximum (FWHM) of the Ar peak (Carroll *et al.*, 2001 ▸), the resolution at 244 eV is then revealed to be 1.8 × 10^−4^ (Δ*E*/*E*) in Fig. 2 ▸(*b*). Similarly, the rocking curve of the DCM at 5000 eV (the Bragg angle θ is 23.3°) gives the FWHM of 82.0 µrad in Fig. 2 ▸(*c*), which corresponds to the energy resolution [Δ*E*/*E* is roughly equal to FWHM × cot (θ)] of 1.9 × 10^−4^ at 5000 eV. Secondly, photon flux has been checked at the endstation via measurement of the photocurrent of the calibrated photodiode (AXUV-100G); this reveals that fluxes of the soft X-ray beam and hard X-ray beam are 3.3 × 10^12^ photons s^−1^ at 300 mA and 244 eV (0.1% bandwidth) and 3.0 × 10^12^ photons s^−1^ at 300 mA and 5.0 keV (0.1% bandwidth), respectively. With a photon flux of the magnitude of 10^12^ photons s^−1^, we can reliably conduct both *in situ* HAXPES and XAS measurements not only for light elements, but also for 3*d* transition metals and heavy elements in the combined endstation. In addition, the beam spot size was measured at 244 eV and 5000 eV by scanning a sharp-edged Au foil horizontally and vertically (step size 1 µm) and recording the corresponding photocurrent (Fig. S2). The spot profile was obtained by fitting the first derivative of the photocurrent curve with a Gaussian function. Consequently, beam sizes are revealed to be: 97.2 µm × 42.1 µm at 244.0 eV, 54.1 µm × 39.5 µm at 5.0 keV, as shown in Figs. 3 ▸(*a*)–3 ▸(*d*).

## 3. The design of the endstation

**Figure 4:**
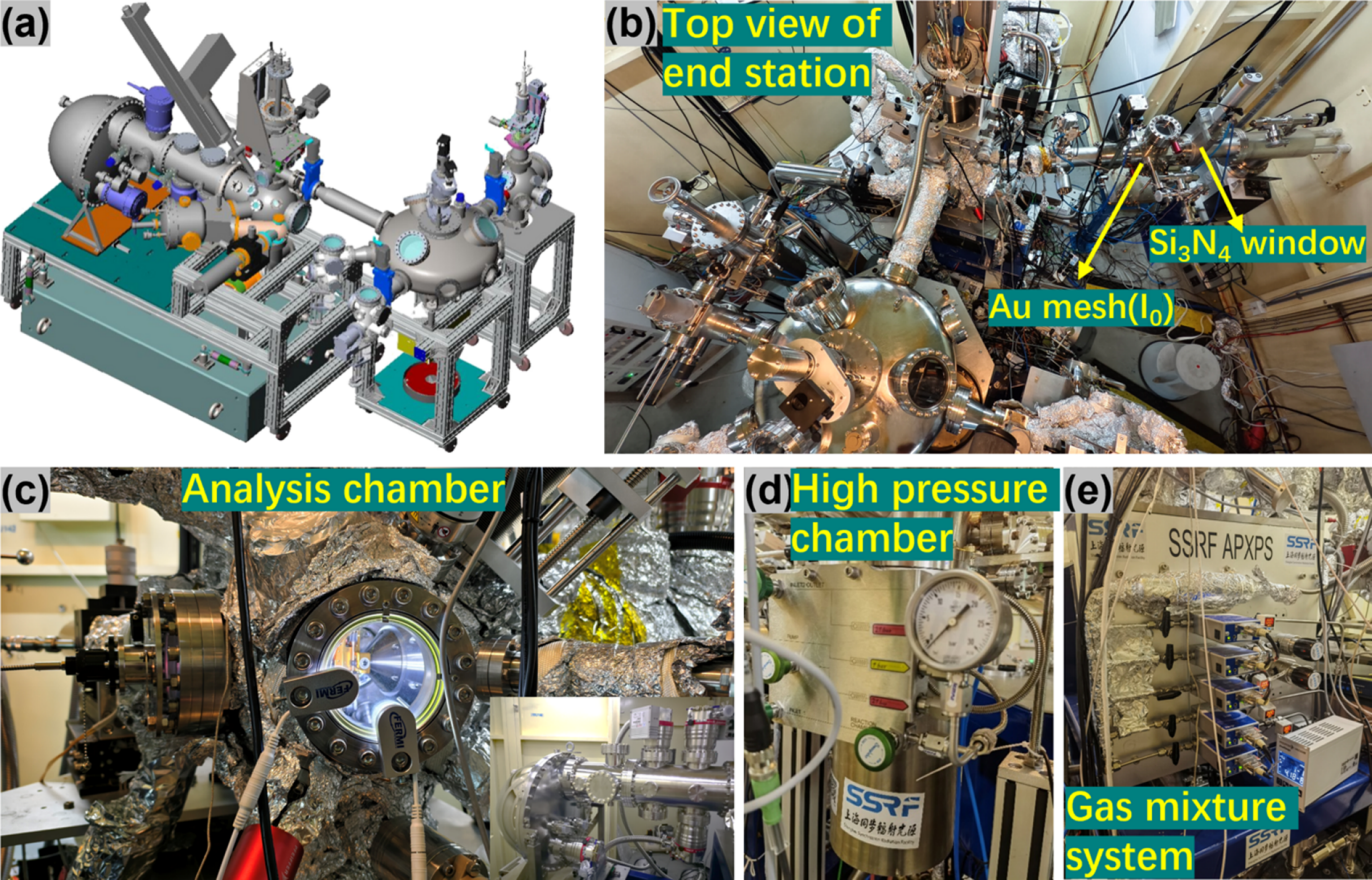
(*a*) Scheme of the combined NAPXPS endstation, which consists of the fast-entry load-lock, the UFO chamber, preparation chambers and the analysis chamber with the laser heating and (*b*) the top view photograph of the NAPXPS endstation. (*c*) The configuration of the sample stage (laser heating from back) and electron analyzer during the ambient-pressure measurement with the inset showing the HIPP-2 electron analyzer integrated in the analysis chamber. (*d*) The high-pressure preparation chamber with gas up to 20 bar and the sample heated to 800°C. (*e*) The gas mixture system for the setup of the solid–gas environment, where five gas lines are introduced and controlled by the automatic control system.

The layout of the endstation is illustrated in Fig. 4 ▸(*a*). As noted previously, this endstation in the combined branch is designed for wide-energy *in situ* XPS and *in situ* XAS studies. Here, *in situ* XPS refers specifically to near-ambient-pressure XPS (NAPXPS), with the current focus on elucidating solid–gas and solid–solid interfaces. Accordingly, the endstation comprises a fast-entry load-lock (FELL), a transfer chamber (UFO), an ultrahigh-vacuum (UHV) preparation chamber, an *operando* preparation chamber (up to 20 bar and 800°C) and an analysis chamber, as illustrated in the top-view schematic in Fig. 4 ▸(*b*). Samples in various forms of powders, thin films or crystal pieces can be mounted on flag-type sample holders and introduced via the load-lock. They may then be transferred to the UHV preparation chamber for further processing before being moved into the analysis chamber [Fig. 4 ▸(*c*)] via the UFO transfer system. Available processing options include *in situ* cleaning by Ar^+^ sputtering, thermal annealing and molecular or thin-film deposition using an electron-beam evaporator or a Knudsen-cell evaporator. Alternatively, samples can be further treated under *operando* conditions in the high-pressure chamber [Fig. 4 ▸(*d*)], such as reduction or oxidation reactions in various gas atmospheres at pressures up to 20 bar and temperatures up to 800°C. The analysis chamber is equipped with a HIPP-2 electron analyzer (ScientaOmicron), a four-axis manipulator and an automated gas-mixing system [Fig. 4 ▸(*e*)] consisting of five mass-flow-controlled gas lines (O_2_, CO_2_, CO, H_2_ and CH_4_). The HIPP-2 spectrometer can precisely detect electrons with kinetic energies from 5 eV to 10000 eV, matching well the photon energy range of the combined branch. Gasses are introduced into the entire analysis chamber via backfilling at pressures up to 100 mbar. The entrance cone of the analyzer can be selected with diameters of 800 µm or 300 µm (or smaller), depending on the required pressure limit. To protect the beamline’s UHV, a 100 nm-thick Si_3_N_4_ window is installed on a CF100 flange in front of the analysis chamber [highlighted in Fig. 4 ▸(*b*)]. Thanks to the three-stage differential pumping system, the vacuum around the microchannel plate (MCP) of the analyzer remains below 10^−9^ mbar, ensuring the stable operation of the MCP (Cai *et al.*, 2019 ▸). For *in situ* heating under gas atmospheres, the laser heating system on the manipulator allows sample temperatures up to 800°C at 50 mbar. Conventional radiative heating is also available on a separate sample stage. Additionally, samples can be cooled with liquid nitro­gen. During XPS measurements, the sample is typically grounded but can also be floated or biased relative to ground, for example, to enable total-electron-yield (TEY) detection for XAS measurements.

Regarding the geometric configuration between the soft/hard X-rays and the electron analyzer, the solution is not straightforward in the present NAPXPS station. As is known, the best geometry for XPS investigations considering the dipole approximation is to keep the magic angle between the electric vector of incoming polarized X-rays and the photoelectron momentum at about 54.7° (Shard, 2020 ▸). However, when it comes to the tender and hard X-ray region, the non-dipole transitions including the dipole–quadrupole contribution, become significant and have to be taken into consideration (Tanuma *et al.*, 2013 ▸; Guillemin *et al.*, 2006 ▸; Isomura *et al.*, 2014 ▸). Of course, the non-dipole effect might also be witnessed in the soft X-ray region in some special cases (Hemmers *et al.*, 2004 ▸). Roughly, it is preferable to keep the angle between the electric field vector of incident X-rays and the electron analyzer close to 90° in the hard X-ray range. This overall approximation could be described as follows:
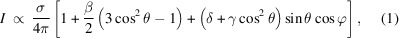
where σ is the total photoionization cross section, β is a dipole asymmetry parameter, and θ is the angle between the incident electric field vector direction of the linearly polarized X-ray and the photoelectron analyzer. This part is the so-called dipole approximation. δ and γ are angular parameters considering electric dipole–quadrupole and electric dipole–magnetic dipole contributions, respectively, while φ is the angle between the photon direction and the plane passing through the photoelectron direction. As seen, the magic angle (54.7°) is valid with the pre-factor of the β parameter in the cross section [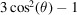] as the major contribution. Taking account of all these factors, the maximum photoelectron intensity at the photon energy of 8000 eV is obtained with θ at about 90° for *s*, *p* states, and differs by 10° or 20° for *d* and *f* states, taking Pt as an example (Isomura *et al.*, 2014 ▸). Notably, the photoelectron intensity at θ of 70° is still about 80% of the maximum intensity at 8000 eV. Meanwhile, in the soft X-ray region, utilizing a geometry larger than the magic angle is also acceptable with the loss of a certain part of the photoelectron intensity, depending on the polarization of X-rays (Shard, 2020 ▸). Consequently, based on these findings and considerations, the angle between the analyzer and hard X-ray is finally set to about 70°, and 68° for the soft X-ray since the angle between the hard X-ray and soft X-ray is about 2°. In most cases, the photon energy will not exceed 8 keV, due to the extremely low photoionization cross section at higher photon energies.

**Figure 5:**
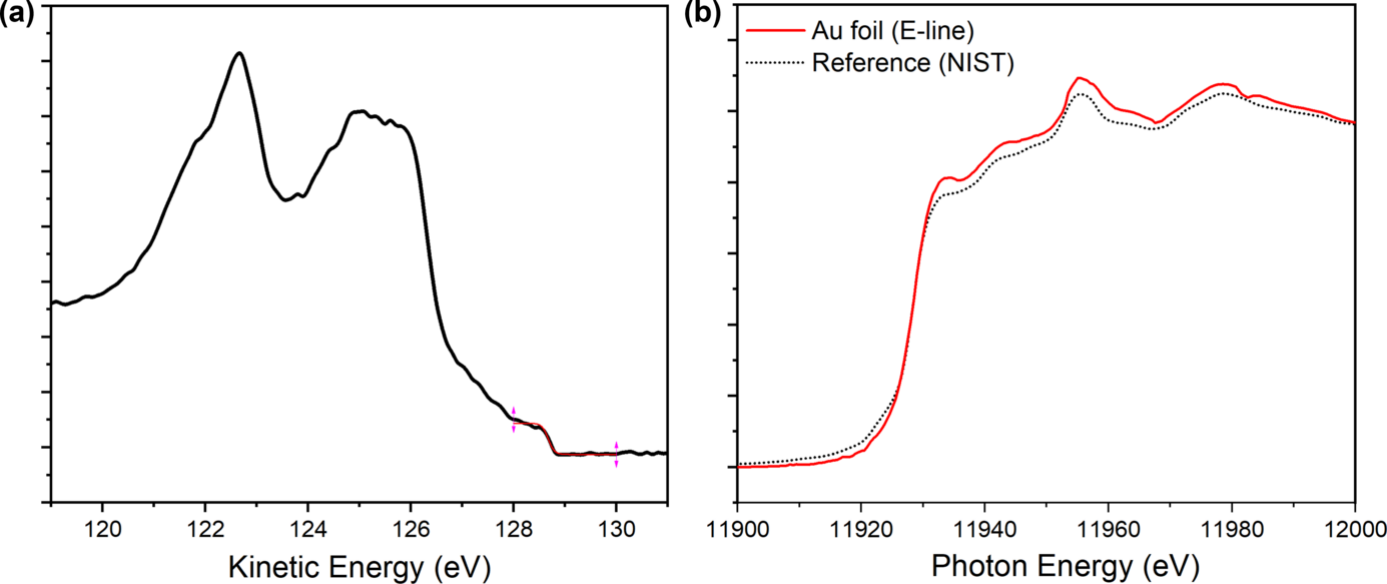
(*a*) Valence band spectroscopy of an Au foil with the Fermi level fitted. (*b*) The Au *L*_3_ absorption spectrum by scanning the incident photon energy and measuring the photoelectric current of the reference sample.

Based on the configuration of the endstation, the available photon energy range of the combined branch was determined by measuring the valence band and the Au *L*_3_ absorption edge of a gold foil, respectively, as shown in Fig. 5 ▸. Fitting the valence band spectrum with a standard Fermi function [Fig. 5 ▸(*a*)] indicates that the lower limit of the photon energy reaches 128.7 eV, slightly below the nominal design value of 130 eV. The observed broadening of the Fermi edge is about 0.06 eV, resulting from the convolution of the intrinsic bandwidth of the incident X-rays, the energy resolution of the electron spectrometer and the thermal smearing of the electron distribution at room temperature. The upper energy limit was established from the Au *L*_3_ absorption edge, recorded by scanning the incident photon energy while using the photocurrent from the sample holder as the *I*_0_ reference. In typical XAS measurements, *I*_0_ is obtained from an Au mesh placed upstream of the endstation; however, this configuration could not be used for the Au *L*_3_ absorption measurement. As shown in Fig. 5 ▸(*b*), the acquired absorption spectrum agrees well with the NIST reference, clearly resolving the absorption edge, confirming that the upper photon energy extends to at least 12 keV. Although the DCM in the combined branch can deliver photons above 12 keV, the photoionization cross section drops significantly at higher energies (Weiland *et al.*, 2016 ▸). Therefore, photons exceeding 10 keV are not currently utilized for research at this endstation.

## 4. *In situ* investigation at the combined soft/hard XPS and XAS spectroscopies endstation

**Figure 6:**
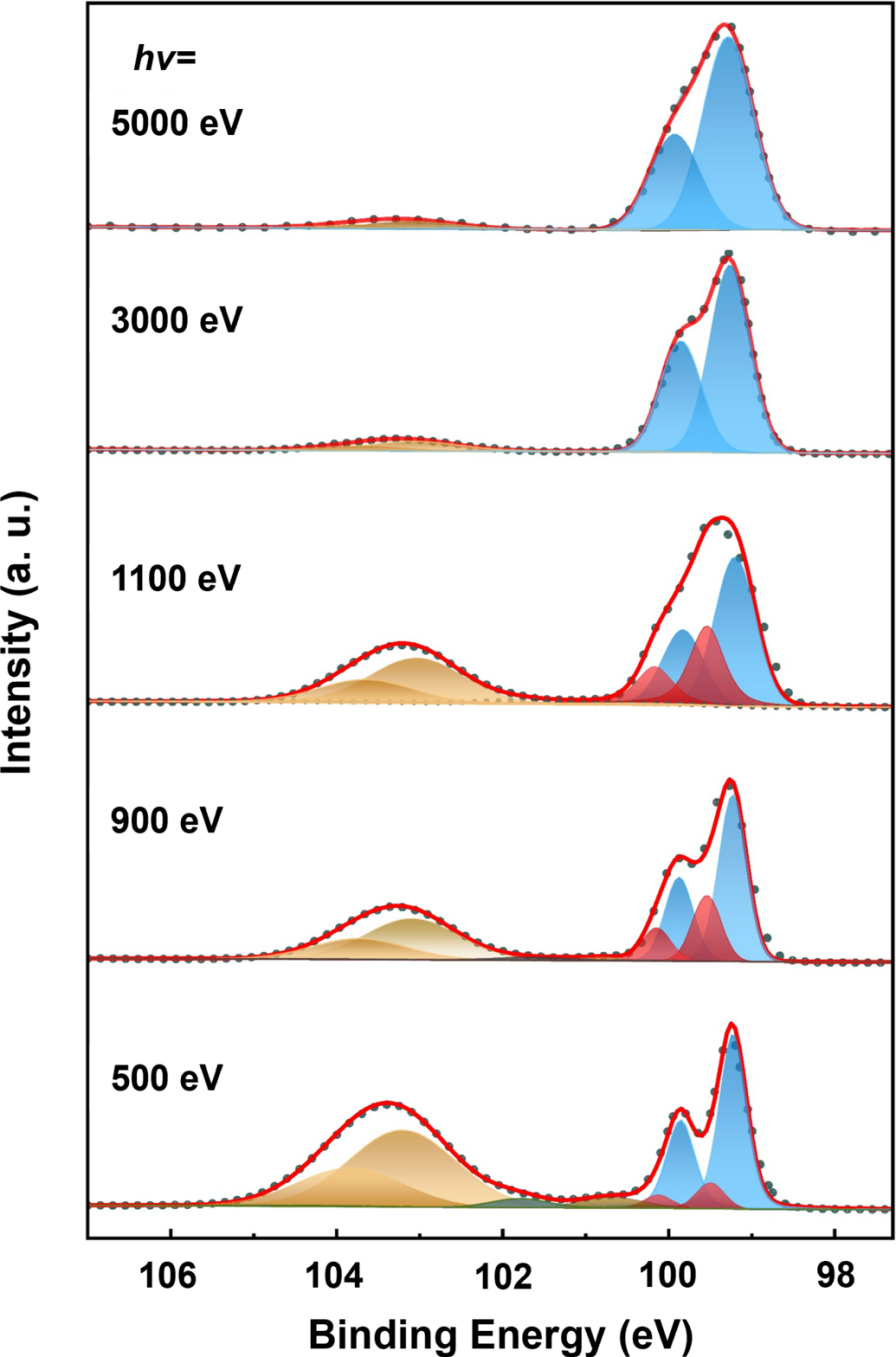
Si 2*p* XPS spectra of the SiO_2_/Si interface at varying photon energies from 500 eV to 5000 eV.

The wide-energy-range XPS capability of the E-line was validated through HAXPES measurements. First, a silicon wafer was cleaned following a standard semiconductor cleaning procedure (Kern, 1990 ▸) and transferred into the analysis chamber for UHV measurements. The corresponding XPS spectra are shown in Fig. 6 ▸, acquired at selected photon energies of 500 eV, 900 eV, 1100 eV, 3000 eV and 5000 eV. At first glance, a paired component is resolved at the binding energy of 103.2 eV for Si 2*p*_3/2_ and 103.9 eV for Si 2*p*_1/2_ with the broadening peak (FWHM around 1.5 eV) (Moulder *et al.*, 1992 ▸), which is assigned to the SiO_2_ layer formed on top of the silicon substrate, as the wafer was not flash-annealed in UHV and such an oxidation layer is commonly observed. While the pristine Si state is discovered at 99.2 eV (Si 2*p*_3/2_) and 99.9 eV (Si 2*p*_1/2_), another doublet appears at 99.5 eV and 100.2 eV, which becomes more distinct at 1100 eV photon energy. This behavior suggests that these paired peaks arise from interface Si atoms, whose chemical state differs slightly from that of bulk atoms (Yamashita *et al.*, 2001 ▸). The hypothesis is further supported by the complete disappearance of these paired peaks at higher photon energies (above 3000 eV). Meanwhile, as expected, the intensity of the oxidation state gradually declines with increasing photon energy and is no longer detectable at 5000 eV, owing to the substantially increased probing depth which enhances sensitivity to bulk layers. Nevertheless, when the photon energy reaches 5000 eV, the intrinsic broadening from X-rays becomes significant (*E*/Δ*E* = 6250 at 5000 eV), which affects the overall resolution of the Si 2*p* spectrum, and the shoulder in the Si 2*p* spectroscopy becomes less visible accordingly.

**Figure 7:**
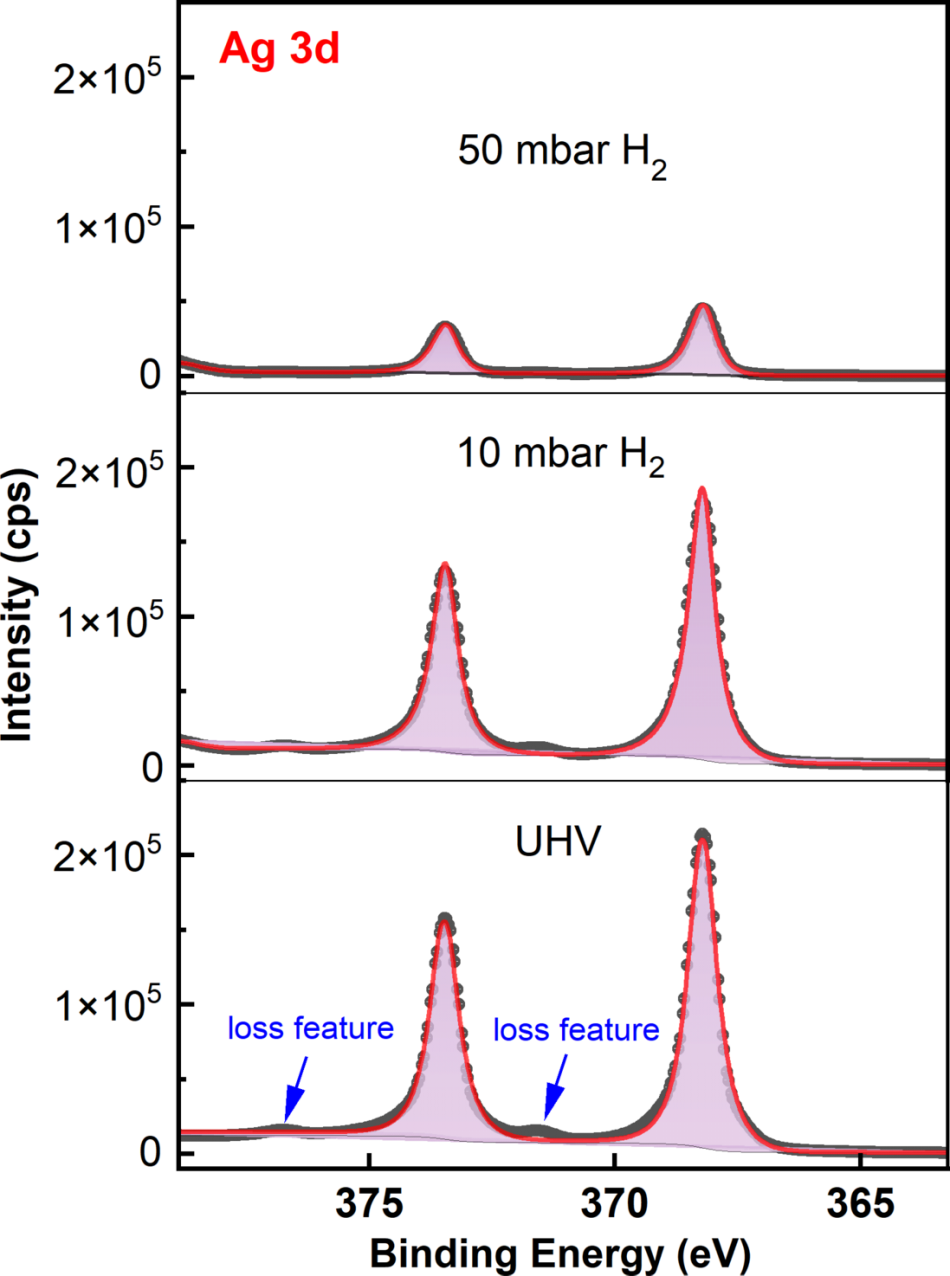
Ambient-pressure XPS investigations of Ag 3*d* core level in the H_2_ atmosphere with *hv* = 1200 eV.

Importantly, to verify the near-ambient condition of XPS in the combined endstation, the solid–gas environment is created by delicately leaking, for instance, H_2_ into the analysis chamber via the leak valve and the automatic gas mixture system, where the pressure can reach 100 mbar with high precision (±1 mbar). In practice, there are two different ways to build the solid–gas environment for *in situ* studies. The first one is called backfilling, namely, filling the whole analysis chamber with the reactant gas. The other way is to utilize a small-volume reactor cell in front of the electron analyzer, and the reactant gas is filled into the cell only. While backfilling is user friendly, the upper pressure limit of backfilling is much lower than for the reactant cell design; however, it is not always feasible to use the reactant cell design. To start with, a clean Ag(111) crystal was prepared via cycles of Ar^+^ sputtering (1000 eV) and post-annealing (700 K), and the cleanness of Ag(111) was checked by XPS with no oxygen or carbon contaminations (Hu *et al.*, 2021 ▸). *In situ* exploration of the Ag 3*d* core level with *hv* = 1200 eV (flux 1.1 × 10^12^ photons ^−1^ at 300 mA and 0.1% bandwidth) in an atmosphere of 10 mbar and 50 mbar H_2_ is presented in Fig. 7 ▸. The robustness of Ag 3*d* XPS to the external H_2_ gas could be identified although the intensity of Ag 3*d* spectrum is reduced considerably in the gas environment (it roughly dropped by 75% at 50 mbar). Moreover, the FWHM of Ag 3*d* peaks is well preserved under *in situ* conditions, while binding energies of both Ag 3*d*_5/2_ and 3*d*_3/2_ components remain unchanged under the H_2_ atmosphere. In addition, the electron loss feature at the higher binding energy side of spin–orbit components, closely related to the metallic Ag state, gets weaker with increasing pressure of H_2_. These *in situ* experiments clearly demonstrate the reliability and feasibility of NAPXPS at the endstation and, in fact, up to 100 mbar could be achieved with the current configuration; even higher pressure is also possible after improvement of the Si_3_N_4_ window, since it is far away from the sample (around 1.5 m) currently. In practice, the backfilling concept is widely adopted at other NAPXPS beamlines worldwide, for instance, at the EMIL beamline in BESSY II (Follath *et al.*, 2013 ▸), where its operation is user friendly. Nevertheless, investigation of the solid–liquid interface will also be possible in the near future with upgrades of the manipulator and chamber (Axnanda *et al.*, 2015 ▸; Zbynek Novotny *et al.*, 2020 ▸), for instance, utilizing a three-electrode setup or the dip–pull approach, while the current research is focused on the solid–gas interface.

**Figure 8:**
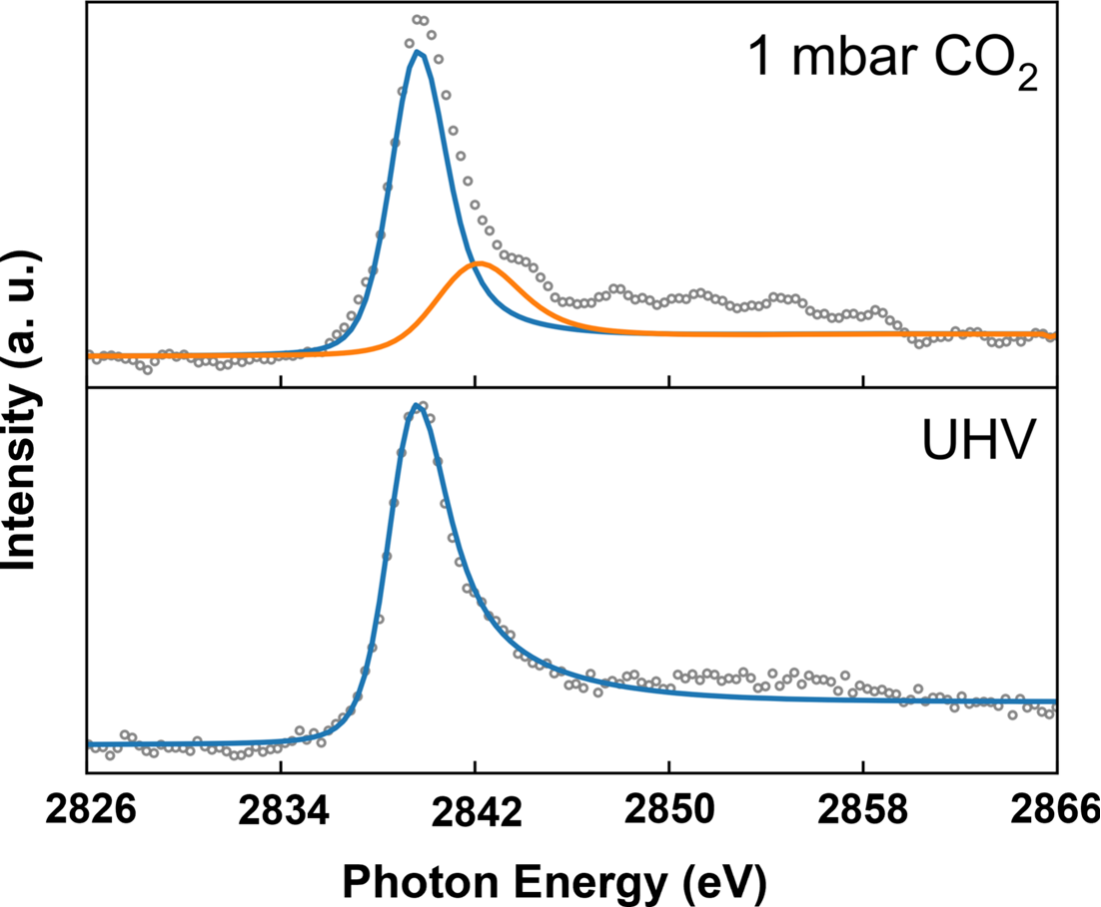
Investigation of the Ru *L*_3_-edge absorption via the TEY mode at UHV and 1 mbar CO_2_.

Beyond studies on single crystals, metal-supported metal thin films with engineered interfacial structures have also been examined for model catalysis research. As an example, the Ru/Cu(111) interface was prepared and investigated using XAS in a CO_2_ environment. Approximately, 1/3 monolayer (ML) Ru was deposited on the Cu(111) substrate via an e-beam evaporator [experimental details are given by Zhang *et al.* (2023 ▸)]. XAS spectra were collected in the TEY mode by measuring the electron current from the sample. First, the Ru *L*_3_-edge absorption spectrum was acquired from the clean Ru/Cu(111) surface. As shown in Fig. 8 ▸, the absorption peak appears around 2840.1 eV, in agreement with earlier reports (Pedersen *et al.*, 2018 ▸). Subsequently, 1 mbar CO_2_ was introduced to the analysis chamber while recording XAS *in situ*. Notably, the TEY signal remained stable under a gas atmosphere with a reasonable signal-to-noise ratio (top panel of Fig. 8 ▸). Apparently, a new feature emerges as a shoulder at approximately 2842.5 eV on the higher-energy side of the main peak (orange curve in Fig. 8 ▸). This additional shoulder can be attributed to oxidized Ru species (Van Kuiken *et al.*, 2013 ▸), indicating that ruthenium undergoes oxidation upon exposure to CO_2_, and the oxidation state likely results from the activation and reduction of CO_2_ at the Ru/Cu(111) interface. The activation of CO_2_ on the Ru/Cu(111) surface at room temperature aligns with previous observations (Zhang *et al.*, 2021 ▸), in which copper-supported metal atoms exhibit pronounced activity towards CO_2_ reduction. Most importantly, these experimental observations clearly confirm the reliability of TEY-based XAS measurements performed under a gas atmosphere in the combined branch of the E-line.

## 5. Conclusions

A wide-energy-range beamline (E-line) comprising soft, hard and combined branches has been constructed at SSRF and user operation began in 2024. By merging soft and hard X-rays into a dedicated combined branch, the beamline offers a specialized platform for wide-energy X-ray spectroscopy, enabling layer-resolved investigation of advanced materials under *in situ* conditions. As demonstrated, the combined endstation can deliver photons from 130 eV to beyond 10 keV via independent insertion devices (IVU and EPU), thereby allowing a comprehensive characterization of model catalysts using near-ambient-pressure XPS (NAPXPS), hard X-ray photoelectron spectroscopy (HAXPES) and X-ray absorption spectroscopy (XAS). This report details the performance of the combined branch and endstation in terms of photon flux, accessible energy range, beam size and representative applications in model catalysis. Equipped with multiple *in situ* spectroscopic techniques, including NAPXPS, HAXPES and TEY–XAS, the combined endstation is poised to substantially advance the fundamental understanding of advanced materials and accelerate progress in renewable energy technologies.

## Supplementary Material

Figure S1 and S2. DOI: 10.1107/S1600577525011506/ok5149sup1.pdf

## Figures and Tables

**Table 1:** Undulator parameters

	No. of periods	Period length (mm)	Length in total (m)	Maxium *k* value	Peak magnetic field (T)	Energy range (eV)
EPU	30	60	1.8	5.3	0.907	130–1500
IVU	65	24	1.56	1.7	0.96	1500–18000

## Data Availability

The data supporting the results reported in the article can be accessed from within the article and upon request.
