# Supplementary material for: A dedicated beamline for wide-energy-range X-ray spectroscopy at SSRF: combining soft and hard X-ray capabilities
Source: J Synchrotron Radiat. 2026 Jan 30;33(Pt 2):523–30. doi: 10.1107/S1600577525011506 (PMC12948002; doi:10.1107/S1600577525011506)
Supplement: Supplementary file 1 [file s-33-00523-sup1.pdf]

JOURNAL OF SYNCHROTRON RADIATION

Volume 33 (2026)

Supporting information for article:

A dedicated beamline for wide-energy-range X-ray spectroscopy at SSRF: combining soft and hard X-ray capabilities

Zhaofeng Liang, Jinyang Xu, Lei Xie, Jingyuan Ma, Bingbao Mei, Liangxin Wang, Nan Wang, Zhenhua Chen, Ying Zou and Fei Song

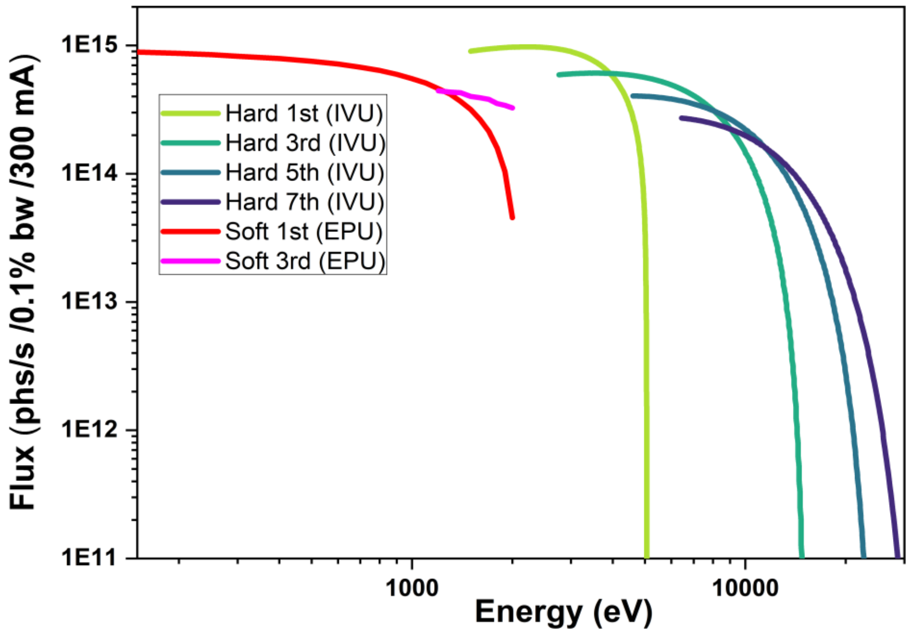

Figure S1 Calculated photon flux of undulators in the first, third, fifth, and seventh harmonic of IVU and in the first and third harmonic of EPU.

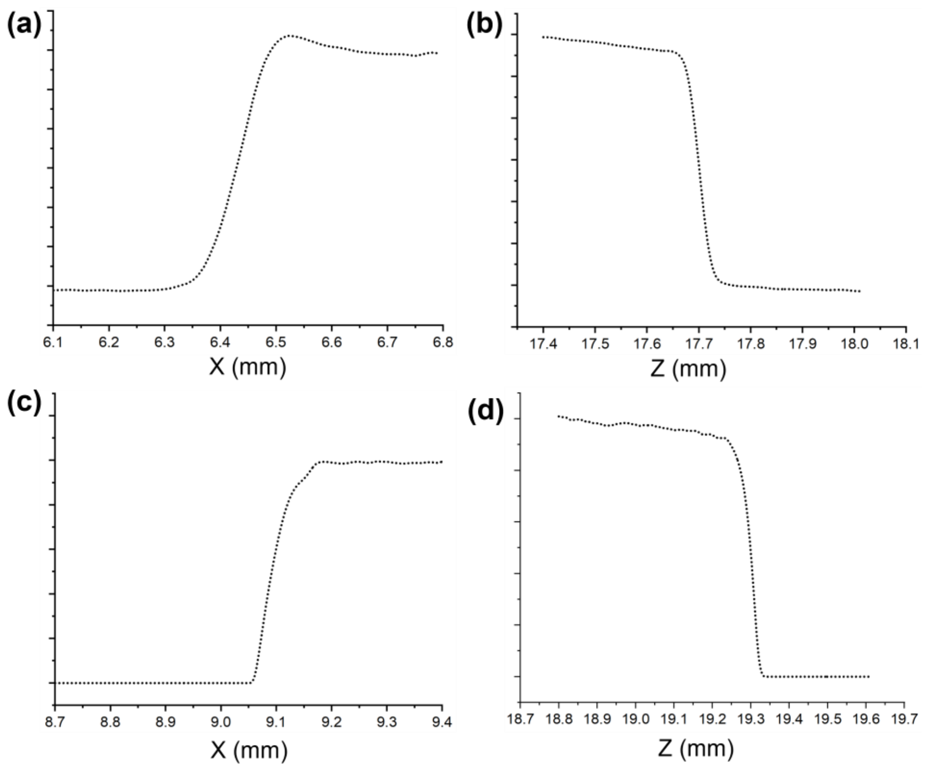

Figure S2 The original photocurrent curves as a function of X/Z movements in correspondence to Fig. 3.
